# Antioxidant Status and Biotechnological Potential of New *Vischeria vischeri* (Eustigmatophyceae) Soil Strains in Enrichment Cultures

**DOI:** 10.3390/antiox12030654

**Published:** 2023-03-06

**Authors:** Aleksandr Yakoviichuk, Zinaida Krivova, Svetlana Maltseva, Angelica Kochubey, Maxim Kulikovskiy, Yevhen Maltsev

**Affiliations:** 1 Faculty of Natural Sciences, Melitopol State University, Melitopol 72312; alex.yakov1991@gmail.com (A.Y.); kochubeya92@yandex.ru (A.K.); 2 Laboratory of Molecular Systematics of Aquatic Plants, K.A. Timiryazev Institute of Plant Physiology RAS, IPP RAS, Moscow 127276, Russia; kosiapeya@mail.ru (Z.K.); svetadm32@gmail.com (S.M.); max-kulikovsky@yandex.ru (M.K.)

**Keywords:** algae, antioxidant activity, carotenoids, chlorophylls, enzymes, fatty acids, phylogeny, vitamins

## Abstract

The functional state of enrichment cultures of the Eustigmatophycean strains *Vischeria vischeri* MZ–E3 and MZ–E4 after 25-day cultivation in the BBM medium was studied. The concentrations of chlorophyll *a*, total carotenoids, protein, vitamins A and E, fatty acid peroxidation product content, an antioxidant enzyme, and succinate dehydrogenase activity were measured. MZ–E3 succinate dehydrogenase activity was significantly higher by 2.21 times; the MZ–E4 strain had 2.94 times higher glutathione peroxidase activity. The MZ–E3 antioxidant activity index and the MZ–E3 unsaturation of fatty acids were 1.3 and 1.25 times higher than the MZ–E4. The retinol and α-tocopherol content of the MZ–E3 was 28.6% and 38.76% higher than MZ–E4. The main fatty acid profile differences were the 3.46-fold and 3.92-fold higher stearic and eicosapentaenoic acid content in the MZ–E4 biomass. MZ–E3 had higher antioxidant, energy, and metabolic and photosynthetic status than MZ–E4. The antioxidant status of the studied strains showed the dependence of the adaptive mechanisms of each, associated with differences in the ecological conditions of the biotopes from which they were isolated. These strains are promising for producing α-tocopherol and biomass enriched with omega-3 and omega-6 fatty acids.

## 1. Introduction

Recently, using microalgae as biotechnological sources of valuable substances is quite common [1,2,3]. Being producers of metabolites with a wide range of biological activity [4], microalgae are used in the production of biodiesel, precursors of medicines, cosmetics, feed additives, and food products [1,3,5,6,7]. However, the issue of finding more productive and unpretentious strains for incubation conditions is still very acute. Considering that stressors of various genesis induce the production of many substances, more resistant strains are regarded as the most promising. It is well known that stress is accompanied by the intensification of lipid peroxidation processes [8,9,10,11,12]. Therefore, a comprehensive study of the state of the antioxidative system (AOS) and the content of various low-molecular-weight antioxidants is a priority in the strategy of selecting strains for their further biotechnological use, and this approach has recently been used by some scientists [13,14]. Such an assessment makes it possible to exclude potentially low-productive strains that synthesise significant amounts of, for example, carotenoids under standard conditions but which are rapidly depleted when the strain is stressed since other branches of AOS have low functional activity. However, this approach does not allow us to assess the type, intensity, and duration of the stressor used to induce the biosynthesis of the necessary substance.

The stability of the cell and the ability to maintain its homeostasis both under physiologically normal conditions and under the influence of stress factors is provided by AOS [12]. Any negative impact and realisation of physiological functions of the body are accompanied by the generation of free radicals, which are intermediaries in the transmission of intracellular signals, the formation of a protective reaction of the body, and in the case of excessive production, the initiation of lipid peroxidation and oxidative modification of biopolymers and metabolites, and the development of oxidative stress, which leads to cell death [15,16,17]. To prevent the result of oxidative modification of cell components, reactive oxygen species (ROS) are neutralised by AOS components, which include enzymes and low-molecular-weight antioxidants and also implement genetically programmed mechanisms to increase oxidative resistance by regulating the metabolism of individual cell components, one of which is a change in the ratio of saturated fatty acids (SFAs) and unsaturated (UFAs) in the composition lipids, as well as a decrease in the intensity of energy metabolism [18,19,20,21,22,23]. Polyunsaturated fatty acids (PUFAs) are the main substrate of peroxide oxidation due to the high affinity of the double bonds of the molecule to ROS [20], and energy supply enzymes are sources of ROS. Therefore, for the comparative characteristics of individual microalgae strains, applying an integrated approach to assessing AOS parameters, the fatty acid composition of the cell, and the energy status is necessary. This approach will make it possible to establish their stability relative to each other and choose a promising strain for biotechnological introduction, based not only on the quantitative content of the target product in a state of the physiological norm and under various types of stress but also on the resistance of the strain to the intensity and duration of exposure to this stressor. The Eustigmatophyceae needs to be better described in terms of screening for the presence of secondary metabolites and their production. The available information is limited mainly to lipids, carotenoids with rare mentions of other vitamin-like substances, and amino acids, and representatives of the genera *Nannochloropsis* D.J. Hibberd and *Vischeria* Pascher are the most studied in this regard [14,24,25,26,27,28,29,30,31,32]. Regarding the study of AOS components and antioxidant resistance, there are some mentions in the work of Assunção et al. [33] for strains of *Vischeria helvetica* (Vischer et Pascher) D.J. Hibberd.

However, considering the information of Goiris et al. [13] about the presence of significant differences in the content of antioxidants even in different strains of the same species, the data presented in the literature are insufficient to establish the patterns of accumulation of metabolites and the formation of an antioxidant response for individual species of Eustigmatophyceae. Let us consider the impact on the biochemical parameters of environmental factors and differences in the composition of the culture medium, temperature, time, and stage of culture growth. The need for in-depth, comprehensive studies of individual species and strains of Eustigmatophyceae becomes apparent. From the point of view of biochemistry, one of the insufficiently studied species is *Vischeria vischeri* (Hibberd) Kryvenda, Rybalka, Wolf et Friedl. There are no references to the content of low-molecular-weight antioxidants and antioxidant resistance of this type in the literature. Considering this information, the goal was to analyse the antioxidant status of *V*. *vischeri* (MZ–E3, MZ–E4) strains based on biochemical markers isolated from different biotopes in the stationary growth phase with prolonged cultivation in a cumulative culture. 

## 2. Materials and Methods

### 2.1. Isolation and Cultivation

The novel strains MZ–E3 and MZ–E4 were isolated by micropipetting from algae enrichment cultures using an inverted Zeiss Axio Vert A1 microscope (Carl Zeiss Microscopy GmbH, Gottingen, Germany). To obtain enrichment cultures, small amounts of soil samples were taken, placed in Petri dishes, and moisturised. Afterwards, the cultures were maintained at room temperature under light intensity of 70 μmol photons m^−2^ s^−1^ and a 16:8 h light/dark photoperiod. Light microscopy observations were carried out using a Zeiss Scope A1 microscope (Carl Zeiss Microscopy GmbH, Gottingen, Germany), equipped with an oil immersion objective (×100, n,a. 1.4, differential interference contrast). Observation of the strain lasted from 24 h to 6 months. The culture was maintained on the Bold Basal Medium (BBM) [34]. The strains were deposited in the Algae Collection of Molecular Systematics of Aquatic Plants at K.A. Timiryazev Institute of Plant Physiology RAS and the Collection of Algae at Melitopol State University CAMU (WDCM1158) as perpetually transferred pure cultures.

**Figure 1:**
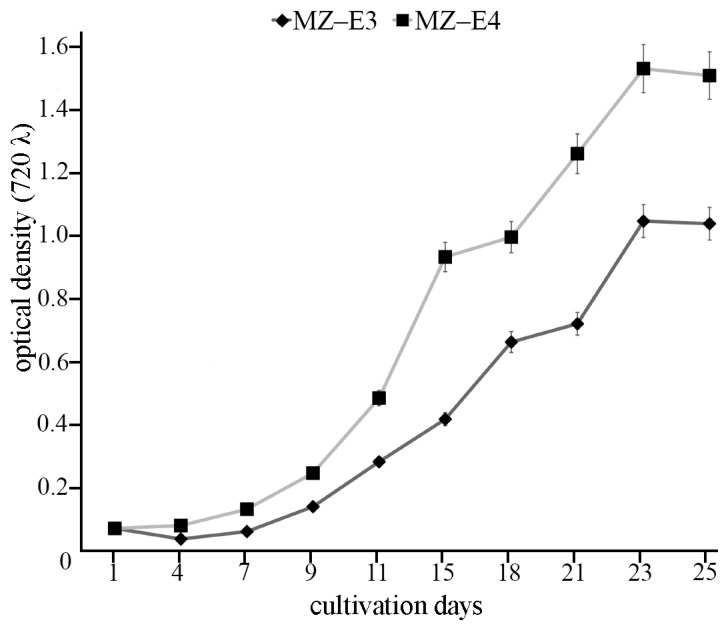
Growth curves of *Vischeria vischeri* MZ–E3 and MZ–E4. Optical density, arithmetic means (M) ± standard errors (S.E.), n = 3.

For biochemical analysis, the cultures were maintained in 250 mL Erlenmeyer glass flasks with 150 mL BBM medium under constant orbital shaking (150 rpm in ELMI Sky Line Shaker S-3 L, ELMI Ltd., Riga, Latvia) for 25 days at 25 °C. The light intensity was 70 μmol photons m^−2^ s^−1^ with colour temperature 4000 K and a 16:8 h light/dark photoperiod. The light intensity and colour temperature were measured using the Sekonic C-800 spectrometer (Sekonic Corporation, Tokyo, Japan). IMPLEN Nanophotometer P300 (Implen GmbH, München, Germany) was used to measure optical density at k = 720 nm (OD_720_). The initial cultures had OD_720_ of 0.063. OD_720_ of 0.9325 corresponds to the stationary growth phase of the strain MZ–E3 on the 25th day of the cultivation. OD_720_ of 1.3558 corresponds to the stationary growth phase of the strain MZ–E4 on the 25th day of the cultivation (Figure 1).

Before the biochemical study, the algal suspensions were conveyed to 15–50 mL tubes (depending on the volume). The cells were pelleted at room temperature for 3 min at 3600 g. The supernatant was removed, and the pelleted cells were resuspended in 10–15 mL (depending on the amount of biomass) of distilled water, quantitatively transferred to 15 mL centrifuge tubes, and pelleted again by centrifugation. The supernatant was removed, and samples were quantitatively transferred to a 50 mL round-bottom flask.

### 2.2. Molecular Analysis

The DNA of the investigated strains MZ–E3 and MZ–E4 was extracted using Chelex 100 Chelating Resin, molecular biology grade (Bio-Rad Laboratories, Hercules, CA, USA), according to the manufacturer’s protocol 2.2. Partial 18S rDNA fragments (444–474 bp, including the highly variable V4 region of the 18S rRNA gene) of algae were amplified using primers D512for and D978rev from Zimmermann et al. [35].

Amplifications were performed using premade polymerase chain reaction (PCR) mastermixes (ScreenMix by Evrogen, Moscow, Russia). Amplification conditions for the V4 region were as follows: initial denaturation for 5 min at 95 °C followed by 35 cycles of 30 s denaturation at 94 °C, 30 s annealing at 52 °C, and 50 s extension at 72 °C, with the final extension for 10 min at 72 °C. The PCR products were visualised by horizontal electrophoresis in 1.0% agarose gel stained with SYBR^TM^ Safe (Life Technologies, Carlsbad, CA, USA). The products were purified with a mixture of FastAP, 10× FastAP Buffer, Exonuclease I (Thermo Fisher Scientific, Waltham, MA, USA), and water. The sequencing was performed using a Genetic Analyzer 3500 instrument (Applied Biosystems, Waltham, MA, USA).

**Figure 2:**
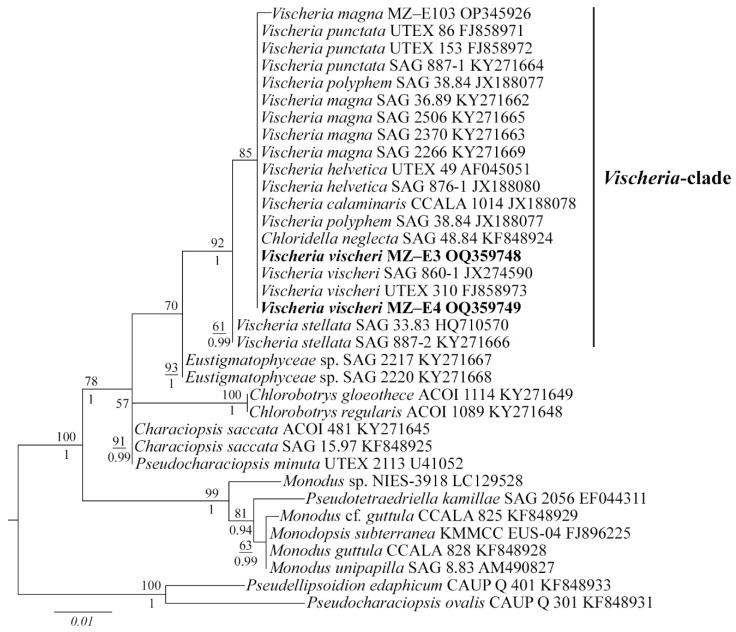
Phylogenetic position of new *Vischeria* strains (indicated in bold) based on Maximum Likelihood from an alignment of 35 sequences and 477 characters (partial 18S rRNA gene). Values above the horizontal lines are bootstrap support from ML analyses (values below 50 are not shown). Values under the horizontal lines are Bayesian posterior probabilities (values below 0.9 are not shown). Strain numbers (if available) and GenBank numbers are indicated for all sequences.

Editing and assembling of the consensus sequences were carried out by processing the direct and reverse chromatograms in Ridom TraceEdit (ver. 1.1.0) (Ridom GmbH, Münster, Germany) and Mega ver. 7 software [36]. The 18S rDNA sequences of the novel strains were included in the alignments of 33 Eustigmatophycean sequences from GenBank (taxa names and Accession Numbers are given in Figure 2). Species from the *Pseudellipsoidion* Neustupa et Nemcová group were chosen as the outgroup. The nucleotide sequences of the 18S rRNA genes were aligned using the Mafft ver. 7 software (RIMD, Osaka, Japan) and the E-INS-i model [37]. The resulting alignments had lengths of 477 characters.

The data set was analysed using the Bayesian inference (BI) method implemented in Beast ver. 1.10.1 software (BEAST Developers, Auckland, New Zealand) [38] to construct a phylogeny. The most appropriate substitution model for the alignment partition, shape parameter α, and a proportion of invariable sites (pinvar) were estimated using the Bayesian information criterion (BIC) as implemented in jModelTest ver. 2.1.10 (Vigo, Spain) [39]. This BIC-based model selection procedure selected the following model and a proportion of invariable sites (pinvar): HKY + I and pinvar = 0.8470. A Yule process tree prior was used as a speciation model. The analysis ran for 5 million generations with chain sampling every 1000 generations. The parameter-estimated convergence, effective sample size (ESS), and burn-in period were checked using the Tracer ver. 1.7.1 software (MCMC Trace Analysis Tool, Edinburgh, UK) [38]. The initial 25% of the trees were removed, and the rest were retained to reconstruct a final phylogeny. The phylogenetic tree and posterior probabilities of its branching were obtained based on the remaining trees, having stable estimates of the parameter models of nucleotide substitutions and likelihood. The maximum likelihood (ML) analysis was performed using RAxML software [40]. The nonparametric bootstrap analysis with 1000 replicas was used. FigTree ver. 1.4.4 (University of Edinburgh, Edinburgh, UK) and Adobe Photoshop CC ver. (19.0) software (Adobe, San Jose, CA, USA) were used for viewing and editing the trees.

### 2.3. Succinate Dehydrogenase Activity

We added 0.9 mL of phosphate buffer (0.1 M pH = 7.8) to 0.1 g of biological material separated from the medium by centrifugation at 3000 rpm for 10 min and then homogenised with quartz sand for 10 min at a temperature of 2–4 °C. The top layer was selected and used as an enzyme source.

To determine succinate dehydrogenase activity (SD) (EC 1.3.99.1), the method of Munjos et al. [41] was used, which is based on the restoration of iodonitrotetrazolium chloride to coloured formazan, which has a maximum absorption at 500 nm. The procedure for determining activity was carried out using a standard Merck kit (Sigma-Aldrich, St. Louis, MO, USA) by the protocol described in the instructions.

### 2.4. Measurement of Chlorophyll Content

The content of chlorophyll *a* (Chl *a*) was determined by the photometric extraction method [42]. Algal biomass (5 mg) was subject to freezing–thawing for partial cell homogenisation. The biomass was then homogenised with 4 mL of 90% acetone (PanReac AppliChem, Barcelona, Spain) with quartz sand, sealed hermetically, and incubated in the dark for 24 h at 25 °C. The extract was separated from the pellet by centrifugation (6000 rpm, 10 min), and measurements were carried out on a Ulab 102 spectrophotometer (Ulab, Nanjing, China) against acetone at 664, 647, and 630 nm, which corresponded to absorption maxima for chlorophylls *a*, *b*, and *c*, respectively. The measurements were carried out in the Multi-wavelength Analysis mode using the MetaSpec Pro ver. 2.2 software package. The content of chlorophylls (mg g^−1^ dry weight (DW)) was calculated according to the following equation [42]:

Chl *a* = 11.85 E_664_ − 1.54 E_647_ − 0.08 E_630_.

### 2.5. Measurement of Carotenoid Content

Carotenoid content was determined by the photometric extraction method [43]. The biomass (5 mg) was subjected to freezing–thawing and homogenised with quartz sand in 4 mL of 100% acetone. The homogenate was incubated for 24 h in the dark in a hermetically sealed vial to extract carotenoids completely. The extract was then separated from the cell debris by centrifugation (6000 rpm, 10 min), and spectrophotometric measurements were taken at 470 nm, i.e., at the maximum carotenoid absorption in 100% acetone. Carotenoid content (mg g^−1^ DW) was calculated according to the following equation [43]:

Car = 1000 A_470_ − 2.270 Chl *a* − 81.4 Chl *b*/227.

### 2.6. Measurement of Vitamin A and E Content

Vitamin content was determined by thin-layer chromatography [44]. Saponification of the algal biomass (30 mg) was carried out in 0.5 N KOH in ethanol (Sigma-Aldrich, St. Louis, MO, USA). For this purpose, 1 mL of the ethanol KOH solution was added to the biomass in a vial, supplemented with 10 mg ascorbic acid (Dia-M, Moscow, Russia), and the mixture was saponified for 30 min at 90 °C. Alcohol forms of retinol and α-tocopherol were retrieved with multi-stage extraction with *n*-hexane (Sigma-Aldrich, St. Louis, MO, USA). Extraction was carried out four times, adding 1.0, 1.0, 1.0, and 0.5 mL *n*-hexane to the hydrolysate; the mixture was left for precipitation, and the hexane extract was then decanted. The extracts were pooled together and washed with distilled water to neutral pH (determined using a universal indicator paper). The extract was then vacuum-evaporated at 55–60 °C. The dry residue was dissolved in 100 µL benzene (PanReac AppliChem, Barcelona, Spain), applied (10 µL) to the starting line of a TLC plate with silica gel 60 (Sigma-Aldrich, USA), and chromatographed. The mobile phase was chloroform (Sigma-Aldrich, USA), and the solvent travelled 8.5 cm. The spots of vitamins were detected using standard solutions. The spots were developed with 1% phosphomolybdic acid (LenReaktiv, Saint Petersburg, Russia) in ethanol; the plates were submerged into the solution for 10 s, dried, and heated for 5 min at 100 °C. The vitamins were revealed as blue spots against the yellow background, decolourised using ammonia vapours for 20 s. The plates were then scanned, and the peak areas and heights were measured using the Sorbflil TLC Videodensitometer ver. 2.3 (JSC Sorbpolymer, Krasnodar, Russian) software package. The concentrations were determined by comparison with the calibration curve built using the standard solutions of retinol and α-tocopherol (Sigma-Aldrich, USA), which were separated and detected on the plate processed under the same conditions as the experimental ones.

### 2.7. Measuring of Antioxidant Enzyme Activity

We added 0.9 mL of phosphate buffer (0.1 M pH = 7.5) to 0.1 g of algal biomass separated from the medium by centrifugation at 3000 rpm for 10 min and homogenised with quartz sand for 10 min at a temperature of 2–4 °C. Further, 0.25 mL of 96% ethanol and 0.15 mL of chloroform were added to 1 mL of the resulting supernatant for partial purification of enzymes and precipitation of ballast proteins. The mixture was stirred for 15 min on a magnetic stirrer in the cold. After the time had elapsed, this mixture was left in the cold for 20 min (0–2 °C), shaking from time to time. The samples were mixed and centrifuged at 10,000 rpm for 15 min. The top layer was selected and used as an enzyme source. A supernatant was used without treatment with ethanol and chloroform to determine the activity of glutathione peroxidase. 

Glutathione peroxidase activity (GPO) (EC 1.11.1.9) was determined by the Moin [45] method. A total of 0.5 mL of 0.25 M tris buffer (pH = 7.4), 0.1 mL 25 mM EDTA, 0.1 mL 0.4 M sodium aside, and 0.05 mL tert-butyl hydrogen peroxide were added to 0.3 mL of the supernatant and incubated for 10 min. The samples were centrifuged for 10 min at 9000 rpm. A total of 0.1 mL of the resulting incubation medium was injected into pre-prepared test tubes containing 5 mL of tris buffer, and 0.1 mL of Elman reagent was added. After 5 min, OD_412_ was measured. We used the following formula to calculate the activity:

A = (OD_CS_ − OD_TS_) (11.4 × 5^−1^ × m^−1^)^–1^,

where m is the protein weight in the sample; OD_CS_ is the optical density of the control sample; OD_TS_ is the optical density of the test sample; 11.4 and 5 are coefficients that take into account the specific molar absorption of the Elman-glutathione reagent complex and the volume of the incubation medium.

Catalase activity (CAT) (EC 1.11.1.6). The method is based on the conversion of hydrogen peroxide by the enzyme and the ability of ammonium molybdate to form a stable-coloured complex with H_2_O_2_ (λ = 410 nm) [46]. The molar extinction coefficient of the aqueous solution of this complex is 22.2 × 10^3^ mol^−1^ cm^−1^.

Superoxide dismutase activity (SOD) (EC 1.15.1.1). The method is based on the ability of SOD to inhibit the reduction process of nitroblue tetrazolium (NBT) under conditions of generation of a superoxide anion radical [47].

### 2.8. Measurement of TBA-Active Product Content

The products of peroxide lipid oxidation are determined by their ability to form a coloured complex with 2-thiobarbituric acid (TBA-active products, TBAaP) upon heating under acidic conditions [48]. The TBA-active products were extracted with 1.2% KCl (PanReac AppliChem, Barcelona, Spain). Algal biomass (0.1 g) was supplemented with 0.9 mL KCl solution and homogenised with quartz sand. Centrifugation separated the supernatant from the sediment (9000 rpm, 15 min).

To determine the TBAaP content in the original homogenate, the extract (0.2 mL) was supplemented with 1.6 mL of the phosphate buffer (0.5 M, pH = 7.35) and 1 of 2-thiobarbituric acid (Sigma-Aldrich, St. Louis, MO, USA) dissolved in glacial acetic acid. The mixture was heated for 60 min at 95 °C. After cooling to room temperature, OD_532_ was measured.

TBAaP content was also determined under the induction of peroxide lipid oxidation (TBAaPin). The reaction mixture containing 0.2 mL homogenate and 1.5 mL phosphate buffer was supplemented with 0.1 mL of 1% FeSO_4_ solution (LenReaktiv, Saint Petersburg, Russia) and incubated for 30 min at 37 °C. TBAaP_in_ was determined as described above. The concentration was calculated according to the following equation:

C = D × 28/31.2,

where C is the TBAaP concentration, nmol g^−1^; D is optical density; and 28 and 31.2 are the coefficients accounting for specific molar absorption of the trimethine complex of TBA with TBA-active products and the dilution factor for the incubation medium volume of 2.8 mL.

### 2.9. Coefficient of Antioxidant Activity and Fatty Acid Unsaturation Calculation

As an integral indicator of the state of the antioxidant protection system, the antioxidant activity coefficient (K_AOA_) was used, which was calculated as the ratio of the content of TBAaP to TBAaP_in_ [49].

K_AOA_ = TBAaP TBAaP_in_^−1^

The total unsaturation (TU) was also calculated: the total equivalent concentration of fatty acids in mmol g^−1^ relative to the number of double bonds, which can act as an indicator of the resistance of lipids of biological membranes to peroxide oxidation. The calculation was carried out according to the following formula [49]:

TU = m n 10^3^ M^−1^,

where m is the mass of UFAs in 1 g of a mixture of fatty acids, g; M is the molar mass of UFAs, g mol^−1^; and n is the number of double bonds in the UFA molecule.

### 2.10. Measurement of Fatty Acid Content

Biomass preparation for determining the fatty acid methyl ester (FAME) profiles was performed according to Maltsev et al. [50]. Heptadecanoic acid (Sigma-Aldrich, St. Louis, MO, USA) was used as the internal standard for fatty acid composition determination. All samples were processed under an argon atmosphere to avoid the oxidation of UFAs. Ten millilitres of a 1 M solution of KOH in 80% aqueous ethanol was added to the dry residue, and the flask was sealed with a reflux condenser and kept for 60 min at the boiling point of the mixture (~80 °C). After the time-lapse, the solvents were evaporated in vacuo to a volume of ~3 mL and quantitatively transferred with distilled water to a 50 mL centrifuge tube to a total volume of 25 mL, followed by extracting the unsaponifiable components with 10 mL portions of *n*-hexane (Himmed, Moscow, Russia) 3 times. To accelerate the separation of the phases, the tube was centrifuged for 5 min at room temperature and 2022× *g*. After that, the aqueous phase was acidified to a slightly acidic reaction (on indicator paper) with a few drops of 20% sulfuric acid (Himmed, Moscow, Russia), and free fatty acids were extracted with 20 mL of *n*-hexane. The hexane solution of free fatty acids was transferred to a dry 50 mL round-bottom flask. The solvent was evaporated to dryness using a rotary evaporator IKA RV-10 (IKA-WERKE, Staufen im Breisgau, Germany), after which 10 mL of absolute methanol (Sigma-Aldrich, St. Louis, MO, USA) and 1 mL of acetyl chloride (Sigma-Aldrich, St. Louis, MO, USA) were added to the dry residue. The flask, closed with a reflux condenser, was kept for one hour at 70 °C; then the solvents were evaporated to dryness, a few drops of distilled water were added to the dry residue, and FAMEs were extracted with *n*-hexane.

The obtained FAMEs were analysed using an Agilent 7890A gas-liquid chromatograph (Agilent Technologies, Santa Clara, CA, USA) with an Agilent 5975C mass spectrometric detector. A DB-23 capillary column 60 m long and 0.25 mm in diameter was used (Agilent Technologies, Santa Clara, CA, USA). The remaining conditions of the analysis were as follows: carrier gas was helium, flow rate of 1 mL min^−1^, 1 μL volume of injected sample, 1:5 flow divider, and the evaporation temperature of 260 °C. Temperature gradient program: from 130 to 170 °C in 6.5 °C min^−1^ steps; from 170 to 215 °C in 2.5 °C min^−1^ increments, 215 °C for 25 min, from 215 to 240 °C in 40 °C min^−1^ increments, and the final stage lasting 50 min at 240 °C. The operating temperature of the mass spectrometric detector was 240 °C, and the ionisation energy was 70 eV.

### 2.11. Measurement of Protein Content

Proteins were determined by the bicinchoninic acid assay [51]. The biomass (10 mg) was freeze-thawed and homogenised with quartz sand in the medium of ethanol (1 mL) to extract and remove chlorophylls and lipophilic substances. The extract was separated by centrifugation (6000 rpm, 10 min), and the pellet was resuspended in 1 mL of the extraction phosphate buffer (0.1 M, pH 7.5, SDS 0.5%, Sigma-Aldrich, United States) and left for 12 h at 25 °C. The calibration graph was obtained using the standard solution of bovine serum albumin (Thermo Scientific, Waltham, MA, USA).

### 2.12. Data Analysis

All measurements were carried out in three repetitions. The graphs show the average values and errors of the average. Statistical data were obtained in Microsoft Excel ver. 1903 (Microsoft Office, Redmond, WA, USA). The reliability of the differences between the indicators was calculated using the Tukey criterion with the Bonferroni correction. The differences at *p* ≤ 0.05 were considered reliable.

## 3. Results

### 3.1. Strain Description

*Vischeria vischeri* MZ–E3 (Eustigmatophyceae).

Morphological description. Mature cells spherical, rarely ovoid, and 5.0–15.0 μm in diameter. Chloroplast parietal, cup-shaped, and slightly incorrectly lobed in mature cells. Reproduction by autospores only (usually 2–4).

Habitat. The strain was isolated from a sample of the upper 5 cm layer of soil, selected in a mixed plantation of Crimean pine (*Pinus pallasiana* D. Don) and black locust (*Robinia pseudoacacia* L.) in the “M. Gorky Melitopol Central Park of Culture and Recreation” (N 46°50′17.04″, E 35°21′46.15″), Melitopol, Zaporizhzhia region, 10 November 2012. The soil is kastanozem; pH (water) of the soil is 5.06; humus content is 6.1%; and ash content is 62.3% [52].

Sequence data. GenBank accession OQ359748 for the 18S rRNA gene partial sequence.

*Vischeria vischeri* MZ–E4 (Eustigmatophyceae).

Morphological description. Mature cells spherical and 3.7–6.5 μm in diameter. Chloroplast parietal, trough-shaped, and slightly incorrectly lobed in mature cells. Reproduction by autospores only (usually 2–4).

Habitat. The strain was isolated from a sample of the upper 5 cm layer of soil, selected in an alder (*Alnus glutinosa* (L.) Gaertn) forest (N 48°45′41.70″, E 35°26′35.69″), Dnipropetrovsk region, November 28, 2012. The soil is gleysol; pH (water) of the soil is 5.4; humus content is 10.5%; and ash content is 70.6%.

Sequence data. GenBank accession OQ359749 for the 18S rRNA gene partial sequence.

### 3.2. Molecular Analysis

The 18S rDNA phylogenetic tree was constructed with 35 nucleotide sequences of closely related Eustigmatophycean taxa derived from the National Center for Biotechnology Information (NCBI) and the sequences of the novel strains. All *Vischeria* strains, including MZ–E3 and MZ–E4, form one separate clade (Figure 2). ML and BI analyses gave congruent results for the phylogenetic placement, supported by high statistical values (Figure 2). *V*. *vischeri* MZ–E3 and MZ–E4 were most similar to authentic strain *Vischeria vischeri* SAG 860-1 and *V*. *vischeri* UTEX 310.

### 3.3. Succinate Dehydrogenase Activity

**Figure 3:**
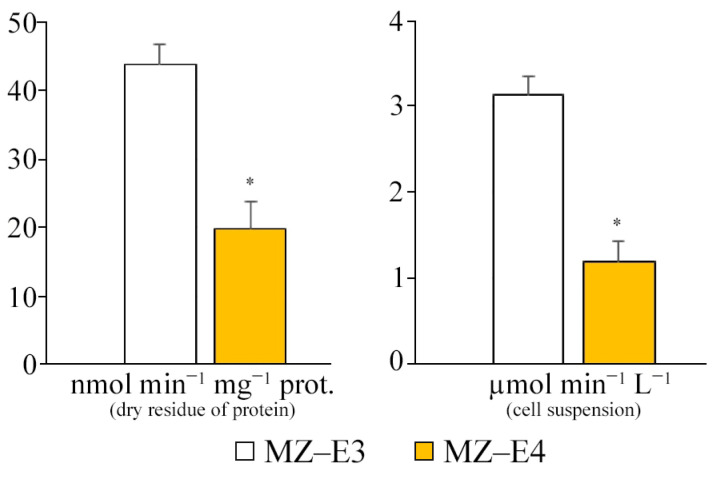
SD activity on the dry residue of protein and cell suspension of *Vischeria vischeri* strains (MZ–E3; MZ–E4). Medium: BBM; culture volume: 250 mL; incubation time: 25 days; M ± S.E., n = 3; * the differences are significant relative to MZ–E3 (*p* ≤ 0.05).

The comparative characteristics of the MZ–E3 and MZ–E4 strains demonstrate a significantly higher SD activity of the MZ–E3 strain by 2.21 times in terms of dry protein residue and 2.61 times in terms of volume (Figure 3). 

### 3.4. Antioxidant Enzyme Activity

**Figure 4:**
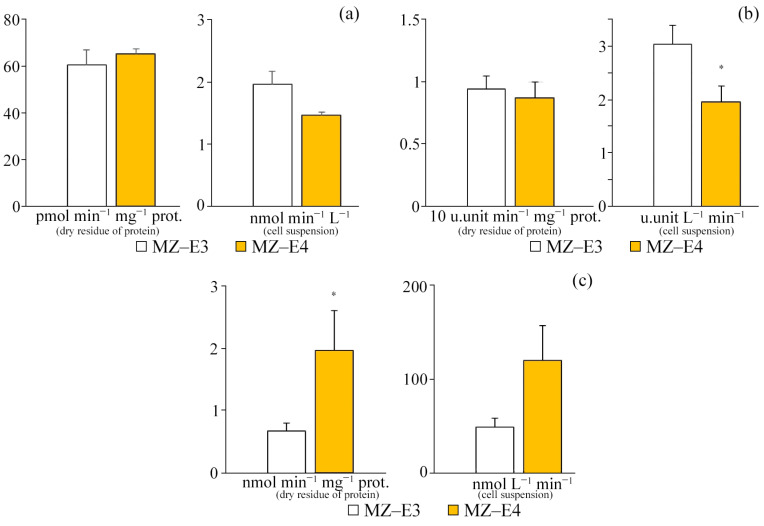
Antioxidant enzyme activity on the dry residue of protein and cell suspension of *Vischeria vischeri* strains (MZ–E3; MZ–E4). (**a**) CAT activity. (**b**) SOD activity. (**c**) GPO activity. Medium: BBM; culture volume: 250 mL; incubation time: 25 days; M ± S.E., n = 3; * the differences are significant relative to MZ–E3 (*p* ≤ 0.05).

For different strains (MZ–E3, MZ–E4) of the *V*. *vischeri*, there is no significant difference in CAT and SOD activity in terms of protein dry residue (Figure 4). At the same time, the MZ–E4 strain has 2.94 times higher GPO activity. When calculating the activity per unit volume of biomass, there was also a significantly higher GPO activity by 2.49 times and a reduced SOD activity by 1.55 times for MZ–E4 relative to MZ–E3.

### 3.5. Products of Lipid Peroxidation

**Figure 5:**
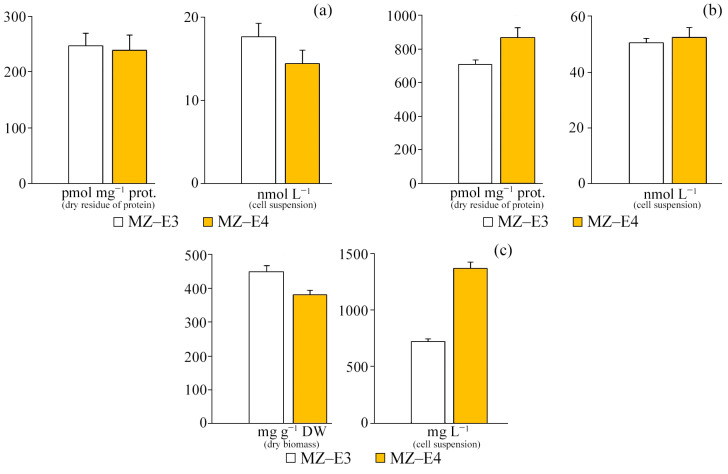
TBA-active products and protein content on the dry residue of protein and cell suspension of *Vischeria vischeri* strains (MZ–E3; MZ–E4). (**a**) TBAaP. (**b**) TBAaP_in_. (**c**) Protein. Medium: BBM; culture volume: 250 mL; incubation time: 25 days; M ± S.E., n = 3.

According to the results of the study, it was found that there are no significant differences in the content of TBA-active products in the initial homogenate and during the initiation of peroxide oxidation by Fe^2+^ ions both in terms of dry residue and in the suspension of cells between the strains (Figure 5). However, it is worth noting that the increased TBAaP_in_ content by 22.7% in the MZ–E4 strain indicates a slightly reduced resistance of this representative to peroxide oxidation. The protein content of MZ–E4 is 3.60 times higher in terms of volume.

### 3.6. Antioxidant Activity Coefficient and Fatty Acid Unsaturation

**Figure 6:**
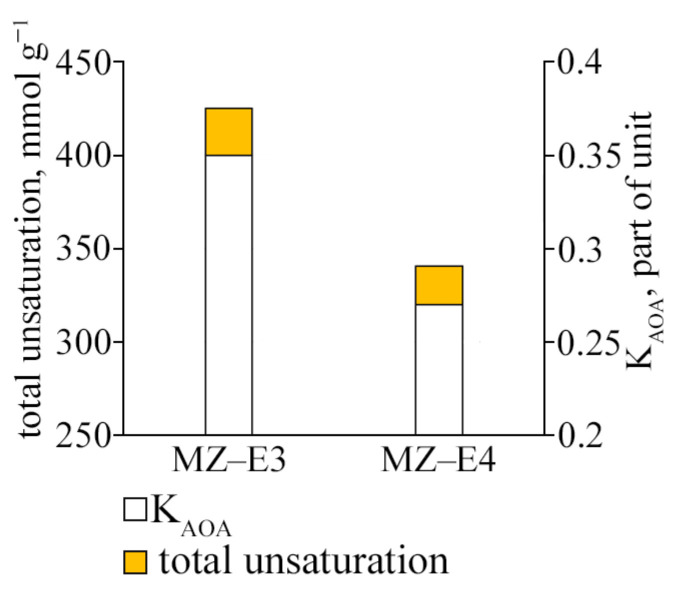
Antioxidant activity coefficient and fatty acid unsaturation of *Vischeria vischeri* strains (MZ–E3; MZ–E4). Medium: BBM; culture volume: 250 mL; incubation time: 25 days; M ± S.E., n = 3.

The study of the antioxidant activity index of strains (K_AOA_) showed that the strain MZ–E3 offers the most significant test resistance to lipid peroxidation. Its coefficient value is 1.3 times higher than MZ–E4 (Figure 6). The resistance to lipid peroxidation is also determined by the unsaturation of the FAs that form them since it is UFAs that are the main substrate of lipid peroxidation. The unsaturation of lipids of the strain MZ–E3 was found to be 1.25 times higher than the corresponding indicator MZ–E4. 

### 3.7. Retinol Content

**Figure 7:**
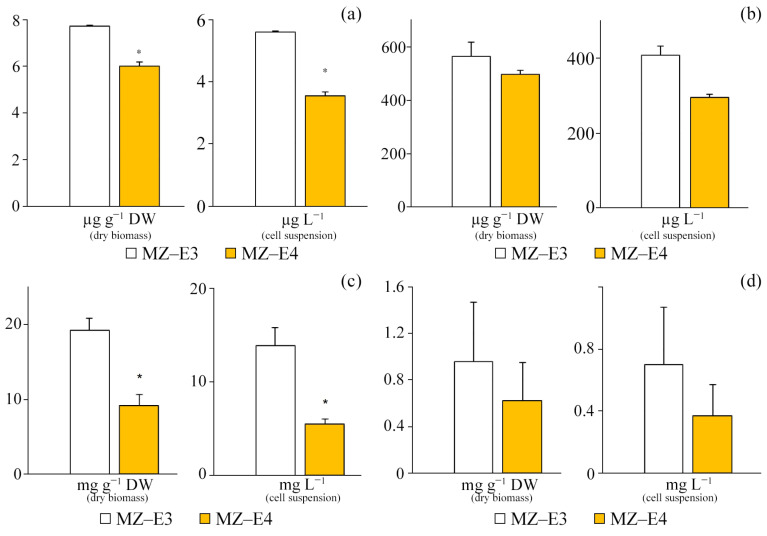
Chlorophyll and secondary metabolites are in the dry biomass and cell suspension of *Vischeria vischeri* strains (MZ–E3; MZ–E4). (**a**) Retinol. (**b**) α-tocopherol. (**c**) Carotenoids. (**d**) Chlorophyll *a*. Medium: BBM; culture volume: 250 mL; incubation time: 25 days; M ± S.E., n = 3; * the differences are significant relative to MZ–E3 (*p* ≤ 0.05).

At the stationary stage of growth, the vitamin A content in the dry residue and suspension of cells of the MZ–E3 strain significantly exceeded this indicator for MZ–E4 by 28.6% and 57.3%, respectively (Figure 7a). 

### 3.8. α-Tocopherol Content

Both studied strains of *V*. *vischeri* are characterised by a high content of α-tocopherol in terms of both volume and dry biomass (Figure 7b). However, significant differences in the secondary metabolite content are observed only for the suspension of cells of the studied strains, where the content of vitamin E in MZ–E3 is 38.76% higher in relation to MZ–E4.

### 3.9. Carotenoid Content

The carotenoid content in the dry residue of the MZ–E3 strain was set at 19.2 ± 7.50 mg g^−1^ DW, which is 2.1 times higher than the corresponding indicator in MZ–E4. When calculating the content of carotenoids per unit volume of biomass, this trend persists, but the difference increases to 2.56 times (Figure 7c).

### 3.10. Chlorophyll Content

The screening of *V*. *vischeri* strains MZ–E3 and MZ–E4 for chlorophyll content demonstrated the presence of only Chl *a* in both cases. The remaining pigments in these strains have yet to be identified (Figure 7d). The Chl *a* content in the dry residue is 54.8% higher in the strain MZ–E3. In the cell suspension, the pigment content for the MZ–E3 strain is also 89.19% higher than MZ–E4. 

### 3.11. Fatty Acid Profile

The analysis of the fatty acid profile of strains MZ–E3 and MZ–E4 demonstrates significant differences in both the qualitative and quantitative composition of the FA, regardless of whether the strains belong to the same species, *V*. *vischeri* (Table 1). The main differences are the 3.46-fold higher content of stearic acid in the MZ–E4 strain and the 3.92-fold higher eicosapentaenoic acid content in MZ–E4. Moreover, the complete absence of hypogenic, vaccenic, and dihomo–γ-linolenic acids was found for MZ–E4. This nature of the composition causes a 105.12% increased content of omega-3 acids in MZ–E3 and 27.74% omega-6 in MZ–E4.

## 4. Discussion

### 4.1. Retinol Content

In microalgae, vitamin A performs antioxidant functions and is a metabolite of β-carrotin [53], and therefore, strains with a high content of it should have a higher functional status of the AOS system, especially in the presence of high concentrations of vitamin E, the antioxidant properties of which increase in the presence of retinol, as was established by Tesoriere et al. [54]. In this case, this is confirmed by the increased antioxidant resistance (K_AOA_) of the MZ–E3 strain compared to MZ–E4, since the concentration of retinol for the former is 28.62% higher. This confirms a more intensive course of secondary metabolism in the MZ–E3 strain or the consumption of the metabolite during the stationary growth phase in the MZ–E4. The absolute concentrations of retinol for the studied strains in terms of dry residue are relatively low, since among the representatives of the Eustigmatophyceae, the retinol content is described only for the genus *Nannochloropsis* and is 50–80 µg g^−1^ [31], which is higher than that of the strains of *V*. *vischeri* studied by us. The relatively low retinol content may be a consequence of the inhibition of the conversion processes of carotenoids, which are its provitamins [53], or the expenditure of the metabolite on the inactivation of active forms of oxygen, and the prevention of the development of oxidative stress during the stationary growth phase.

### 4.2. α-Tocopherol Content

In the literature, there are isolated mentions of the content of α-tocopherol for representatives of the Eustigmatophyceae. In particular, in the works of Mudimu et al. [29], Safafar et al. [14,28], and Del Mondo et al. [31], the content of α-tocopherol for representatives of this class was established in an extensive range of 20.0–4720.0 µg g^−1^. In particular, for two strains of *Vischeria stellata* (Chodat) Pascher (SAG 33.83, SAG 887-2), the vitamin E content is 40.81 and 45.17 µg g^−1^ [29]. If we compare the results obtained in our study with the data of Mudimu et al. [29], the vitamin E content is in the concentration range set at the level of the Eustigmatophyceae. However, it is 13.0 and 11.56 times higher than the corresponding indicator for representatives of the genus of strains MZ–E3 and MZ–E4. Perhaps this is due to the stationary stage of microalgae growth. Mudimu et al. [29] also noted a 39.8% increase in the content of α-tocopherol in the stationary stage compared to the logarithmic for *V*. *stellata* (SAG 887-2), which is associated with the activation of secondary metabolism in the stationary growth phase [29]. A high vitamin E content may indicate an increased resistance of strains to external factors that lead to the activation of peroxide oxidation since vitamin E performs antioxidant functions and prevents the development of oxidative stress [31]. However, the content of this metabolite should be considered in combination with the concentrations of retinol and carotenoids, which are synergistic with vitamin E, increasing its antioxidant properties [54,55]. Taking into account the increased retinol concentration for MZ–E3 at equal concentrations of α-tocopherol, as well as a higher value of K_AOA_, the increased antioxidant resistance of the MZ–E3 strain is confirmed. In addition, retinol plays a significant role and possibly a central role in providing antioxidant protection for *V*. *stellata* species since a significant decrease in the content of this particular secondary metabolite leads to a 29.63% decrease in the coefficient of antioxidant activity of MZ–E4, with a significantly unchanged concentration of α-tocopherol.

### 4.3. Carotenoid Content

Carotenoids are low-molecular-weight antioxidants and inactivate ROS [56]. Accordingly, the accumulation of carotenoids is one factor that increases the cell’s antioxidant status and its resistance [13]. The content of carotenoids for representatives of the Eustigmatophyceae varies in the range of 10.0–80.0 mg g^−1^ depending on the strain’s type and time and conditions of cultivation [14,24,25,28,57,58]. However, for the most-described in the literature species *V*. *helvetica*, the content of carotenoids varies quite widely from 1.0–25.4 mg g^−1^ under physiological conditions to 80 mg g^−1^ under culture stress. Within the genus *Vischeria* (*V*. *helvetica*, *Vischeria punctata* Vischer, *V*. *stellata*), the content of these secondary metabolites is 10.0–80.0 mg g^−1^ [25,57,58]. The strains of the species *V*. *vischeri* studied by us fit into the range of concentrations of carotenoids characterising the genus *Vischeria*. As noted earlier, the carotenoid content of the MZ–E3 strain is higher than that of MZ–E4. This causes the high antioxidant status (K_AOA_) of the first strain. After all, it is known that carotenoids exhibit antioxidant properties and, according to Del Mondo et al. [31], have more potent antioxidant properties than other intracellular antioxidants. At the same time, Stahl et al. [55] report an increase in the antioxidant activity of α-tocopherol in the presence of carotenoids, which again confirms the increased antioxidant status of cells of the MZ–E3 strain compared to MZ–E4. In addition, if we take into account that the concentration of both synergists (retinol, carotenoids) of α-tocopherol is higher for the MZ–E3 strain, the significant contribution of vitamin A and carotenoids to the formation of antioxidant resistance (K_AOA_) of the studied species (*V*. *vischeri*) is fully confirmed. Considering this fact, it can be assumed that strains of this species, regardless of their place of origin, consider implementing an antioxidant response by the same mechanism but with different sensitivity to the strength of stress and incubation time. These strains are not suitable for the production of carotenoids and retinol since it is these substrates that are the first to be consumed to maintain oxidative processes at the level necessary for life support, even though the content of α-tocopherol does not change, and probably this substrate is involved in the formation of an antioxidant response at the last moment, even after the implementation of alternative AOS mechanisms (reduction of unsaturation; slowing down aerobic oxidation). This makes the strains of the *V*. *vischeri* species convenient for obtaining vitamin E in biotechnological conditions since, probably, the intensity and time of exposure to the stress factor, having wide limits, do not affect the consumption of the metabolite. It is worth noting that in the case of the biotechnological production of α-tocopherol, the strain MZ–E4 realises its potential as quickly as possible since, with equal initial parameters and incubation time of the strains, the onset of the depletion of intracellular antioxidants and the start of a decrease in FA unsaturation occurs earlier for it.

### 4.4. Chlorophyll Content

The functional activity of a plant organism is primarily determined by the intensity of photosynthetic processes that provide a general metabolism aimed at energy supply and the synthesis of secondary metabolites. Accordingly, the chlorophyll content determines the activity of the photosynthetic apparatus. It demonstrates a reaction to the negative impact of various factors, which is especially significant when comparing strains of the same species from different habitats, since this allows one, in combination with other biochemical parameters, to evaluate the mechanisms of adaptation of the organism to various conditions and assess the stability of each of the strains when grown in laboratory conditions. 

The screening of *V*. *vischeri* strains MZ–E3 and MZ–E4 for the content of chlorophylls *a*, *b*, and *c* demonstrated the presence in both cases of only Chl *a*, and the remaining pigments in these cultures were not identified, which is confirmed by previously published works for representatives of the Eustigmatophyceae [14,25,28,57]. The Chl *a* content is higher in the MZ–E3 strain, demonstrating the cell’s photosynthetic potential. It suggests an increased intensity of the production of secondary metabolites, which is partially confirmed by the increased concentration of retinol and carotenoids in the cells of the strain, most of which are synthesised only during photosynthesis [59]. Against the background of these results, there is an increased content of retinol, α-tocopherol, and carotenoids in the strain MZ–E3 compared to MZ–E4, which is very important at the stationary stage of growth, which occurs under unfavourable conditions and is accompanied by the accumulation of substances with cytoprotective activity. The lower pigment content in MZ–E4 is probably due to the reduced activity of enzymes responsible for its biosynthesis [60] or the increased activity of reductases responsible for its utilisation, which is more likely because, in the conditions of the stationary growth phase, the decay of chlorophyll serves as one of the mechanisms for providing the cell with nutrients [61] and precursors for the biosynthesis of substances with cytoprotective activity. The total content of Chl *a* for the studied strains is below, described for representatives of the Eustigmatophyceae, and is in the range of 1.07–9.89 mg g^−1^ DW for the following species: *V*. *helvetica* 3.9–9.89 mg g^−1^; *V*. *punctata* 1.9 mg g^−1^; *V*. *stellata* 2.1 mg g^−1^; *Microchloropsis salina* (D.J. Hibbard) M.W. Fawley, I. Jameson et K.P. Fawley 2.00 mg g^−1^; and *Nannochloropsis limnetica* L. Krienitz, D. Hepperle, H.-B. Stich et W. Weiler 1.06 mg g^−1^ [14,28,57]. The different phases of crop growth can explain such deviations since Li et al. [24,25] used an 18-day-old culture at the logarithmic stage of growth; in our case, the age of the crops is 25 days, and both strains are in the stationary phase. Moreover, in this case, it is worth noting the genetic characteristics of the strains and the determining influence of environmental factors on the content of chlorophylls. The spread of the pigment content for different representatives of *Vischeria* may differ by 2.0 times, and for strains of the same species *V*. *vischeri,* isolated from different biotopes, the difference is 54.8%.

### 4.5. Fatty Acid Profile

Fatty acids, performing structural, energy, and regulatory functions, are one of the essential compounds that ensure the physiologically normal functional state of the cell and also act as a marker of its metabolic state [62,63]. In addition, as noted earlier, the regulation of the saturated–unsaturated FA ratio in the composition of cellular structures ensures the adaptive function of the cell to peroxide oxidation and the stress factor that provoked it. In this case, the study of the FA composition of the cell allows us to assess its functional state, the stage of growth, and the nature of the implementation of protective functions. 

The analysis of literature sources indicates significant differences in the composition of FAs among representatives of the Eustigmatophyceae, in both qualitative and quantitative composition, which depends not only on the generic and species affiliation but also on the duration and conditions of culture cultivation and growth stage. It determines differences even within strains of the same species [28,32,64,65]. 

Studies conducted by Sinetova et al. [66] of the strain IPPAS H–242 (*V*. *punctata*) demonstrate significant similarity in the main components of the FA profile with MZ–E3 and MZ–E4. The more remarkable similarity is shown by MZ–E3, while MZ–E4 has a 4.0-time lower content of eicosapentaenoic and a 2.0-time higher content of arachidonic acids, both concerning the described strain of IPPAS H–242 and concerning MZ–E3. At the same time, the screening of the FA composition of strains MZ–E3 and MZ–E4 at the stationary growth stage shows significant differences within the same species (*V*. *vischeri*). This is due to the specifics of the formation of the antioxidant response by the MZ–E4 strain and the restructuring of metabolism to reduce the unsaturation of FA lipids. It is known that the antioxidant response of algae cells at the level of lipid metabolism is realised by reducing the concentration of PUFAs [8], which in this case occurs due to the conversion of polyunsaturated eicosapentaenoic acid. The 20:5n-3 conversion pathway can be provided either by enzyme systems with preliminary chain elongation under the action of elongates, desaturases Δ^4^ and Δ^6^, and subsequent β-oxidation [20,67] or is a process of oxidative acid modification under the action of ROS. The first mechanism is initiated a second time through activating specific factors by free radicals or lipid breakdown products as an antioxidant response [8], and the second is a consequence of the excessive accumulation of ROS.

Moreover, a significant decrease in the content of eicosapentaenoic acid compared to MZ–E3 may be a process of the oxidative modification of the acid under the action of ROS and further decomposition. In addition, it is worth noting that the accumulation of 16:1n-9 and 18:1n-7 for MZ–E3 indicates the activation of enzymes (Δ^9^–desaturase) of the hypogeic acid biosynthesis site (16:1n-9) from palmitic acid (16:0), the Stearoyl-CoA desaturase–2 system, Elovl5, Elovl6 [20], and the enzymes Elovl5 and Δ^6^, Δ^8^–desaturases responsible for the conversion of linoleic and linolenic acids into dihomo-γ-linolenic acid [5,20]. MZ–E4 probably has increased enzyme activity (Δ^5^–desaturase) of the 20:3n-6 conversion site to arachidonic acid [26], which is indirectly indicated by the depletion of the dihomo-γ-linolenic acid pool. MZ–E4 probably has increased enzyme activity (Δ^5^–desaturase) of the 20:3n-6 conversion site to arachidonic acid [67], which is indirectly indicated by the depletion of the dihomo-γ-linolenic acid pool. In general, it is worth noting that the established differences in the composition of FAs between *V*. *vischeri* strains are due to an increase in the antioxidant resistance of the cell in response to unfavourable conditions of the stationary growth stage, primarily a lack of nutritional components. It can be assumed that a decrease in the content of eicosapentaenoic acid in MZ–E4, if it is realised through β-oxidation, provides two functions: providing the cell with energy against the background of a decrease in photosynthetic activity and the activity of the key enzyme of the Krebs cycle (SD) and the implementation of an increase in cell resistance to the action of ROS. In this case, this particular way of reducing the concentration of eicosapentaenoic acid in MZ–E4 is the most preferable.

### 4.6. Succinate Dehydrogenase Activity

Succinate dehydrogenase, in addition to being an essential component of the tricarboxylic acid cycle and the electron transfer chain, providing energy metabolism and energy accumulation, is a source of superoxide and hydrogen peroxide [68,69,70,71], which, with excessive generation, cause oxidative stress, and with moderate generation, are important signalling molecules that ensure the regulation of metabolic process cells [72]. In addition, the enzymes of the tricarboxylic acid cycle are important components of the AOS of the cell since their activity is regulated not only according to the classical feedback scheme but also through alternative mechanisms, providing, if necessary, a decrease in the production of ROS, the sources of which they are. The increased SD activity in the strain MZ–E3 indicates an intensive course of energy processes, even though the generation of superoxide and hydrogen peroxide is either at a constitutive level or at a lower level than that of MZ–E4, as evidenced by the reduced activity of enzymes of the AOS system. At the same time, SD activity is lower for MZ–E4 against the background of increased GPO activity, which indicates the accumulation of organic peroxides and hydrogen peroxide in the cell. In this case, it can be unequivocally stated that there is an inhibition of SD since, as Manhas et al. [72] have established, the generation of hydrogen peroxide by the FAD site of SD is possible only as a result of its inhibition and the unavailability of fumarate. The mechanism of enzyme activity reduction itself may be realised by its deglutationylation, which is associated with the activation of GPO and the consumption of its central coenzyme, glutathione, since it is known about a significant increase in SD activity in the glutathione-acylated state [69]. This mechanism can be considered one of the ways to increase antioxidant resistance and reduce the sensitivity of the enzyme itself to oxidative stress [73].

The very difference in SD activity between the strains suggests that the metabolic potential of MZ–E3 and antioxidant resistance is higher than that of MZ–E4. The increased SD activity indicates the intensive course of anaerobic reactions that provide the body’s energy processes, which may be associated with a more intensive secondary metabolism than MZ–E4. This is confirmed by a significant accumulation of protein, retinol, α-tocopherol, the sum of carotenoids, and long-chain polyunsaturated FAs compared to MZ–E4. Since SD is one of the critical enzymes of the Krebs cycle and, simultaneously, a component of the electron transport chain, it ensures the flow of aerobic metabolism by providing the cell with energy in the form of ATP. Accordingly, this substrate is necessary to ensure energy-intensive processes of the biosynthesis of primary and secondary metabolites.

### 4.7. Antioxidant Enzyme Activity

Antioxidant defence enzymes provide detoxification of free radicals and primary products of the oxidative modification of lipids, which is aimed at preventing the development of oxidative stress [74,75]. Changes in the activity of AOS enzymes can serve as a marker of the result of oxidative stress and the type of reactive products generated and can be used to assess the state of the cell. The activity of SOD, a vital enzyme of the AOS system [76], did not differ between strains. This may indicate the controlled production of a superoxide radical and its further dismutation by the enzyme to hydrogen peroxide. It is worth noting that in the MZ–E4 strain, the regulation of superoxide generation is partially provided by the inhibition of SD activity, which is one of its primary sources [71], confirmed by a 2.21-fold decrease in SD activity. However, as described above, the inhibition of SD activity causes the accumulation of hydrogen peroxide and probably organic peroxides, which causes a 2.49-fold increase in GPO activity in the MZ–E4 strain. Moreover, the decrease in SD activity against the background of increased GPO activity may be caused by both the initial inhibition of SD with further accumulation of peroxides and activation of GPO and the initial generation of peroxide by alternative metabolic systems of the cell with further activation of GPO, the functioning of which leads to the depletion of glutathione reserves and deglutationylation of SD, which reduces its activity. At the same time, the CAT activity for the studied strains is set at the same level, which looks somewhat strange with the alleged accumulation of hydrogen peroxide. However, it is known that GPO has a greater affinity for hydrogen peroxide than catalase, although it is a selenium-containing enzyme that provides detoxification of hydrogen peroxide and lipid hydroperoxides. Its functioning requires a specific coenzyme, glutathione [77]. Accordingly, GPO plays an additional role in the AOS for autotrophic organisms due to the need for an energy-intensive synthesis of glutathione or regeneration of its oxidised form. In this case, it can be assumed that catalase is inhibited in the MZ–E4 strain by superoxide, SOD is inhibited by peroxide [76], and GPO provides prevention of the development of oxidative stress at the level of high-molecular antioxidants. In general, the established changes indicate the accumulation of a significant amount of organic peroxides and hydrogen peroxide both in the cell and in the biomass of the MZ–E4 strain and also indicate an increased generation of superoxide radicals in the MZ–E3 biomass.

### 4.8. Content of Thiobarbituric Acid Reactive Substances and Proteins

The concentration of TBA-active products in the cell indicates the state of peroxide processes, the intensification of which and the subsequent development of oxidative stress are accompanied by the accumulation of secondary lipid breakdown products. This generally reflects the state of the AOS since the generated ROS attacks lipid fatty acids and accumulates TBA-active products and primary lipid oxidation products (diene conjugates, peroxides, organic free radicals, etc.) [78,79,80]. Minor differences in the content of TBA-active products during the initiation of lipid peroxidation, recorded between strains MZ–E3 and MZ–E4, indicate a slightly reduced antioxidant status of MZ–E4. This is confirmed by a relatively low K_AOA_ value and increased GPO activity. Accordingly, the development of oxidative stress is observed in the MZ–E4 culture, probably caused by a lack of nutrients in the medium, which is characteristic of the stationary growth stage [59]. Accordingly, its further deepening may lead to an even more significant accumulation of secondary products in the cell, which will significantly worsen the quality of biomass and will also be accompanied by the depletion of the pool of secondary metabolites with antioxidant activity, which may eventually lead to ROS-induced apoptosis and cell death [81]. In this comparison, MZ–E3 has a more stable AOS system and a more flexible adaptive apparatus of the cell. Perhaps this is due to the initial environmental factors because the high and stable soil moisture and nutrient availability, characteristic of the initial habitat conditions of the MZ–E4 strain, significantly contrasts with the arid habitat conditions of the MZ–E3 strain in soils with poor nutrients. In addition, the protein content of MZ–E4 was reduced by 17.71%, probably due to the oxidative modification of proteins and FAs and further formation of lipid-amide adducts against the background of developing oxidative stress [15,82], as well as enhanced processes of protein catabolism against the backdrop of a decrease in the availability of nutrients in the environment and a reduction in the intensity of aerobic metabolism due to a decrease in the activity of SD.

### 4.9. Antioxidant Activity Coefficient and Fatty Acid Unsaturation

Analysis of the K_AOA_ of the studied strains indicates an increased antioxidant status of MZ–E3 relative to MZ–E4. Such differences are primarily due to the increased content of low-molecular-weight antioxidants (retinol, carotenoids, α-tocopherol). At the same time, a significantly increased TU of fatty acids was found for MZ–E3, indicating a high antioxidant status of culture cells since fatty acids are the main substrate of lipoperoxidation [16,20,83]. The increased functional activity of the AOS system ensures the quenching of free radicals, which contributes to the intensive flow of energy processes, as indicated by the increased activity of SD, which generates free radicals in the functioning process. Accordingly, MZ–E4 has a reduced antioxidant status of cells, as indicated by K_AOA_ and a decrease in unsaturation, which in this context can be considered either as an alternative mechanism for increasing cell resistance to peroxide oxidation or as a consequence of oxidative modification of fatty acids as a result of increased peroxide processes [20,80]. Accordingly, it is worth noting that prolonged exposure to factors triggering the processes of lipoperoxidation will more quickly lead to the depletion of intramolecular antioxidants and a significant decrease in the content of UFAs in the lipid composition of cells of the strain MZ–E4 compared with the more stable MZ–E3. It is also worth noting that as a result of the use of MZ–E3 for the biotechnological production of UFAs, it is worth considering the implementation of the AOS mechanism through a decrease in the unsaturation of FA lipids. As a result, the strength and duration of the stress factor should not lead to a significant intensification of the processes of lipid peroxidation but be in such a range that the neutralisation of ROS by AOS enzymes and low-molecular-weight antioxidants is realised, but alternative mechanisms are not triggered (the reduction of unsaturation, the intensity of energy metabolism, etc.).

## 5. Conclusions

The increased antioxidant, energy, and metabolic and photosynthetic status of the strain MZ–E3 in relation to MZ–E4, strains of the *V*. *vischeri*, has been experimentally established. When growing crops under identical conditions, the process of forming an antioxidant response in MZ–E4 to unfavourable factors in the stationary growth stage occurs earlier than in MZ–E3. It is formed by reducing the content of low-molecular antioxidants (retinol, carotenoids), rearranging lipid metabolism aimed at lowering the PUFA content, activating glutathione peroxidase, and reducing the reactive oxygen species production by reducing the activity of succinate dehydrogenase. The antioxidant status of the studied strains of *V*. *vischeri* shows dependence on the adaptive history of each of them, associated with differences in the ecological conditions of the biotopes from which they were isolated. During laboratory cultivation, the antioxidant response of the MZ–E4 strain isolated from a biotope with more stable and favourable growth conditions, compared with the MZ–E3 strain, in which initial habitat conditions are characterised by extremity, is accompanied by the rapid depletion of adaptive potential. Further research in this direction will make it possible to clarify this regularity, including for strains of other species. Based on the results, it can be stated that these strains are promising for the production of α-tocopherol and biomass enriched with omega-3 and omega-6 fatty acids. In the biotechnological output of α-tocopherol, the strain MZ–E4 realises its potential faster since with equal initial parameters and time of growing strains, the onset of depletion of intracellular antioxidants and the start of lowering the unsaturation of fatty acids for it occurs earlier. When using MZ–E3 for the biotechnological production of unsaturated fatty acids, it is worth considering the implementation of the antioxidative system mechanism by reducing the unsaturation of fatty acid lipids and selecting the strength and duration of the stressor effect. Both strains are not suitable for the production of carotenoids and retinol.

## Figures and Tables

**Table 1:** Fatty acid profile of *Vischeria vischeri* strains.

FA Code	FA Name	MZ–E3		MZ–E4	
		ω, %	U, mmol g^−1^	ω, %	U, mmol g^−1^
14:0	Myristic acid	2.61 ± 0.18	–	2.14 ± 0.15	–
16:0	Palmitic acid	20.21 ± 1.41	–	22.45 ± 1.57	–
18:0	Stearic acid	1.15 ± 0.08	–	3.98 ± 0.28 *	–
16:1n-9	Hypogeic acid	1.70 ± 0.12	6.69	–	–
16:1n-7	Palmitoleic acid	45.07 ± 3.15	177.44	47.88 ± 3.35	188.50
18:1n-9	Oleic acid	15.22 ± 1.07	53.97	14.74 ± 1.03	52.27
18:1n-7	Vaccenic acid	0.70 ± 0.05	2.48	–	–
18:2n-6	Linoleic acid	2.26 ± 0.16	16.14	2.68 ± 0.19	19.14
18:3n-3	alpha-Linolenic acid	2.18 ± 0.15	23.53	2.98 ± 0.21	32.16
20:3n-6	Dihomo-γ-linolenic acid	0.06 ± 0.04	0.59	–	–
20:4n-6	Arachidonic acid	0.60 ± 0.01	7.89	1.05 ± 0.07 *	13.82
20:5n-3	Eicosapentaenoic acid	8.24 ± 0.58	136.42	2.1 ± 0.15 *	34.77
total SFAs		23.97		28.57	
total MUFAs		62.69		62.62	
total PUFAs		13.34		8.81	
total omega-3		10.42		5.08	
total omega-6		2.92		3.73	
ω3:ω6		3.57		1.36	
total Uns.			425.16		340.66

Medium: BBM; culture volume: 250 mL; incubation time: 25 days; M ± S.E., n = 3; * the differences are significant relative to MZ–E3 (*p* ≤ 0.05).

## Data Availability

Data is contained within the article.

## References

[1] Smerilli A., Orefice I., Corato F., Olea A.G., Ruban A.V., Brunet C. (2016). Photoprotective and antioxidant responses to light spectrum and intensity variations in the coastal diatom *Skeletonema marinoi*. Environm. Microbiol..

[2] Maltsev Y., Maltseva I., Maltseva S., Kociolek J.P., Kulikovskiy M. (2021). A new species of freshwater algae *Nephrochlamys yushanlensis* sp. nov. (Selenastraceae, Sphaeropleales) and its lipid accumulation during nitrogen and phosphorus starvation. J. Phycol..

[3] Santin A., Russo M.T., Ferrante M.I., Balzano S., Orefice I., Sardo A. (2021). Highly valuable polyunsaturated fatty acids from microalgae: Strategies to improve their yields and their potential exploitation in aquaculture. Molecules.

[4] Gong Y., Miao X. (2019). Short Chain Fatty acid biosynthesis in microalgae *Synechococcus* sp. PCC 7942. Mar. Drugs.

[5] Martins D.A., Custódio L., Barreira L., Pereira H., Ben-Hamadou R., Varela J., Abu-Salah K.M. (2013). Alternative sources of n-3 long-chain polyunsaturated fatty acids in marine microalgae. Mar. Drugs.

[6] Santos-Sánchez N.F., Valadez-Blanco R., Hernández-Carlos B., Torres-Ariño A., Guadarrama-Mendoza P.C., Salas-Coronado R. (2016). Lipids rich in ω-3 polyunsaturated fatty acids from microalgae. Appl. Microbiol. Biotechnol..

[7] Maltsev Y., Maltseva I., Maltseva S., Kociolek J.P., Kulikovskiy M. (2019). Fatty acid content and profile of the novel strain of *Coccomyxa elongata* (Trebouxiophyceae, Chlorophyta) cultivated at reduced nitrogen and phosphorus concentrations. J. Phycol..

[8] Sun X.M., Geng L.J., Ren L.J., Ji X.J., Hao N., Chen K.Q., Huang H. (2018). Influence of oxygen on the biosynthesis of polyunsaturated fatty acids in microalgae. Bioresource Technol..

[9] Alishah Aratboni H., Rafiei N., Garcia-Granados R., Alemzadeh A., Morones-Ramírez J.R. (2019). Biomass and lipid induction strategies in microalgae for biofuel production and other applications. Microb. Cell. Fact..

[10] Shi T.Q., Wang L.R., Zhang Z.X., Sun X.M., Huang H. (2020). Stresses as first-line tools for enhancing lipid and carotenoid production in microalgae. Front. Bioeng. Biotechnol..

[11] Ajitha V., Sreevidya C.P., Sarasan M., Park J.C., Mohandas A., Singh I.S.B., Puthumana J., Lee J.S. (2021). Effects of zinc and mercury on ROS-mediated oxidative stress-induced physiological impairments and antioxidant responses in the microalga *Chlorella vulgaris*. Environ. Sci. Pollut. Res..

[12] Ren Y., Sun H., Deng J., Huang J., Chen F. (2021). Carotenoid production from microalgae: Biosynthesis, salinity responses and novel biotechnologies. Mar. Drugs.

[13] Goiris K., Muylaert K., Fraeye I., Foubert I., De Brabanter J., De Cooman L. (2012). Antioxidant potential of microalgae in relation to their phenolic and carotenoid content. J. Appl. Phycol..

[14] Safafar H., Van Wagenen J., Møller P., Jacobsen C. (2015). Carotenoids, phenolic compounds and tocopherols contribute to the antioxidative properties of some microalgae species grown on industrial wastewater. Mar. Drugs.

[15] Kato Y., Osawa T. (2010). Detection of lipid-lysine amide-type adduct as a marker of PUFA oxidation and its applications. Arch. Biochem. Biophys..

[16] Arai H., Kato Y. (2014). Oxidative modification of lipoproteins. Lipid Hydroperoxide-Derived Modification of Biomolecules. Subcellular Biochemistry.

[17] Chokshi K., Pancha I., Ghosh A., Mishra S., Kumar M., Ralph P. (2017). Oxidative stress-induced bioprospecting of microalgae. Systems Biology of Marine Ecosystems.

[18] Mailloux R.J., Singh R., Brewer G., Auger C., Lemire J., Appanna V.D. (2009). Alpha-ketoglutarate dehydrogenase and glutamate dehydrogenase work in tandem to modulate the antioxidant alpha-ketoglutarate during oxidative stress in *Pseudomonas fluorescens*. J. Bacteriol..

[19] McLain A.L., Szweda P.A., Szweda L.I. (2011). α-Ketoglutarate dehydrogenase: A mitochondrial redox sensor. Free Radical Res..

[20] Naudí A., Jové M., Ayala V., Portero-Otín M., Barja G., Pamplona R. (2013). Membrane lipid unsaturation as physiological adaptation to animal longevity. Front. Physiol..

[21] Nemeria N.S., Ambrus A., Patel H., Gerfen G., Adam-Vizi V., Tretter L., Zhou J., Wang J., Jordan F. (2014). Human 2-oxoglutarate dehydrogenase complex E1 component forms a thiamin-derived radical by aerobic oxidation of the enamine intermediate. J. Biol. Chem..

[22] Quinlan C.L., Goncalves R.L., Hey-Mogensen M., Yadava N., Bunik V.I., Brand M.D. (2014). The 2-oxoacid dehydrogenase complexes in mitochondria can produce superoxide/hydrogen peroxide at much higher rates than complex I. J. Biol. Chem..

[23] Kurutas E.B. (2016). The importance of antioxidants which play the role in cellular response against oxidative/nitrosative stress: Current state. Nutr. J..

[24] Li Z., Ma X., Li A., Zhang C. (2012). A novel potential source of β-carotene: *Eustigmatos* cf. polyphem (Eustigmatophyceae) and pilot β-carotene production in bubble column and flat panel photobioreactors. Bioresour. Technol..

[25] Li Z., Sun M., Li Q., Li A., Zhang C. (2012). Profiling of carotenoids in six microalgae (Eustigmatophyceae) and assessment of their β-carotene productions in bubble column photobioreactor. Biotechnol. Lett..

[26] Zhang J., Wan L., Xia S., Li A., Zhang C. (2013). Morphological and spectrometric analyses of lipids accumulation in a novel oleaginous microalga, *Eustigmatos* cf. polyphem (Eustigmatophyceae). Bioprocess Biosyst. Eng..

[27] Martin G.J., Hill D.R., Olmstead I.L., Bergamin A., Shears M.J., Dias D.A., Kentish S.E., Scales P.J., Botté C.Y., Callahan D.L. (2014). Lipid profile remodeling in response to nitrogen deprivation in the microalgae *Chlorella* sp. (Trebouxiophyceae) and *Nannochloropsis* sp. (Eustigmatophyceae). PLoS ONE.

[28] Safafar H., Hass M.Z., Møller P., Holdt S.L., Jacobsen C. (2016). High-EPA biomass from *Nannochloropsis salina* cultivated in a flat-panel photo-bioreactor on a process water-enriched growth medium. Mar. Drugs.

[29] Mudimu O., Koopmann I.K., Rybalka N., Friedl T., Schulz R., Bilger W. (2017). Screening of microalgae and cyanobacteria strains for α-tocopherol content at different growth phases and the influence of nitrate reduction on α-tocopherol production. J. Appl. Phycol..

[30] Wang F., Huang L., Gao B., Zhang C. (2018). Optimum production conditions, purification, identification, and antioxidant activity of violaxanthin from microalga *Eustigmatos* cf. polyphem (Eustigmatophyceae). Mar. Drugs.

[31] Del Mondo A., Smerilli A., Sané E., Sansone C., Brunet C. (2020). Challenging microalgal vitamins for human health. Microb. Cell Fact..

[32] Remias D., Nicoletti C., Krennhuber K., Möderndorfer B., Nedbalová L., Procházková L. (2020). Growth, fatty, and amino acid profiles of the soil alga *Vischeria* sp. E71.10 (Eustigmatophyceae) under different cultivation conditions. Folia Microbiol..

[33] Assunção M.F.G., Amaral R., Martins C.B., Ferreira J.D., Ressurreição S., Santos S.D., Varejão J.M.T.B., Santos L.M.A. (2017). Screening microalgae as potential sources of antioxidants. J. Appl. Phycol..

[34] Bischoff H.W., Bold H.C. (1963). Phycological Studies IV. Some Soil Algae from Enchanted Rock and Related Algal Species.

[35] Zimmermann J., Jahn R., Gemeinholzer B. (2011). Barcoding diatoms: Evaluation of the V4 subregion on the 18S rRNA gene, including new primers and protocols. Org. Divers. Evol..

[36] Kumar S., Stecher G., Tamura K. (2016). MEGA7: Molecular evolutionary genetics analysis version 7.0 for bigger datasets. Molec. Biol. Evol..

[37] Katoh K., Toh H. (2010). Parallelization of the MAFFT multiple sequence alignment program. Bioinformatics.

[38] Drummond A.J., Rambaut A. (2007). BEAST: Bayesian evolutionary analysis by sampling trees. BMC Evol. Biol..

[39] Darriba D., Taboada G.L., Doallo R., Posada D. (2012). jModelTest 2: More models, new heuristics and parallel computing. Nat. Meth..

[40] Stamatakis A., Hoover P., Rougemont J. (2008). A rapid bootstrap algorithm for the RAxML web–servers. Syst. Biol..

[41] Munujos P., Coll-Cantí J., González-Sastre F., Gella F.J. (1993). Assay of succinate dehydrogenase activity by a colorimetric-continuous method using iodonitrotetrazolium chloride as electron acceptor. Anal. Biochem..

[42] Jeffrey S.W., Humphrey G.F. (1975). New spectrophotometric equations for determining chlorophylls a, b, c1 and c2 in higher plants, algae and natural phytoplankton. Biochem. Physiol. Pflanzen..

[43] Dere Ş., Güneş T., Sivaci R. (1998). Spectrophotometric determination of chlorophyll—A, B and total carotenoid contents of some algae species using different solvents. Turk. J. Bot..

[44] Hossu A.-M., Radulescu C., Ilie M., Balalau D., Magearu V. (2006). Qualitative and semiquantitative TLC analysis of vitamins A, D and E. Rev. Chim..

[45] Moin V.M. (1986). Prostoĭ i spetsificheskiĭ metod opredeleniia aktivnosti glutationperoksidazy v éritrotsitakh [A simple and specific method for determining glutathione peroxidase activity in erythrocytes]. Lab. Delo.

[46] Góth L. (1991). A simple method for determination of serum catalase activity and revision of reference range. Clin. Chim. Acta.

[47] Fried R. (1975). Enzymatic and non-enzymatic assay of superoxide dismutase. Biochimie.

[48] Zeb A., Ullah F. (2016). Simple Spectrophotometric Method for the determination of thiobarbituric acid reactive substances in fried fast foods. J. Anal. Methods Chem..

[49] Danchenko O.O., Nicolaeva Y.V., Koshelev O.I., Danchenko M.M., Yakoviichuk O.V., Halko T.I. (2021). Effect of extract from common oat on the antioxidant activity and fatty acid composition of the muscular tissues of Geese. Regul. Mech. Biosyst..

[50] Maltsev Y., Krivova Z., Maltseva S., Maltseva K., Gorshkova E., Kulikovskiy M. (2021). Lipid accumulation by *Coelastrella multistriata* (Scenedesmaceae, sphaeropleales) during nitrogen and phosphorus starvation. Sci. Rep..

[51] Olson B. (2016). Assays for determination of protein concentration. Curr. Protoc. Pharmacol..

[52] Maltsev Y., Maltseva A., Maltseva S. (2021). Differential Zn and Mn sensitivity of microalgae species from genera *Bracteacoccus* and *Lobosphaera*. Environ. Sci. Pollut. Res..

[53] Kato Y., Kato Y. (2014). The formation of lipid hydroperoxide-derived amide-type lysine adducts on proteins: A review of current knowledge. Lipid Hydroperoxide-Derived Modification of Biomolecules. Subcellular Biochemistry.

[54] Galván I. (2017). Evidence of evolutionary optimization of fatty acid length and unsaturation. J. Evol. Biol..

[55] Toti E., Chen C.-Y.O., Palmery M., Valencia D.V., Peluso I. (2018). Non-provitamin A and provitamin A carotenoids as immunomodulators: Recommended dietary allowance, therapeutic index, or personalized nutrition?. Oxidative Med. Cell. Longev..

[56] Tesoriere L., Bongiorno A., Pintaudi A.M., D’Anna R., D’Arpa D., Livrea M.A. (1996). Synergistic interactions between vitamin A and vitamin E against lipid peroxidation in phosphatidylcholine liposomes. Arch. Biochem. Biophys..

[57] Stahl W., Heinrich U., Jungmann H., Sies H., Tronnier H. (2000). Carotenoids and carotenoids plus vitamin E protect against ultraviolet light-induced erythema in humans. Am. J. Clin. Nutr..

[58] Maoka T. (2019). Carotenoids as natural functional pigments. J. Nat. Med..

[59] Aburai N., Ohkubo S., Miyashita H., Abe K. (2013). Composition of carotenoids and identification of aerial microalgae isolated from the surface of rocks in mountainous districts of Japan. Algal Res..

[60] Aburai N., Abe K. (2015). Metabolic switching: Synergistic induction of carotenogenesis in the aerial microalga, *Vischeria helvetica*, under environmental stress conditions by inhibitors of fatty acid biosynthesis. Biotechnol. Lett..

[61] Sharma R., Chahar O.P., Bhatnagar M., Bhatnagar A. (2013). Impact of osmotic stress and temperature on pigments and proteins of *Anabaena* strains. J. Environm. Biol..

[62] Margulis B.A., Gushova I.V. (2009). Dual role of chaperones in the response of a cell and of a whole organism to stress. Cytology.

[63] De Carvalho C.C.C.R., Caramujo M.J. (2018). The various roles of fatty acids. Molecules.

[64] Casares D., Escribá P.V., Rosselló C.A. (2019). Membrane Lipid Composition: Effect on membrane and organelle structure, function and compartmentalization and therapeutic avenues. Int. J. Mol. Sci..

[65] Krienitz L., Wirth M. (2006). The high content of polyunsaturated fatty acids in *Nannochloropsis limnetica* (Eustigmatophyceae) and its implication for food web interactions, freshwater aquaculture and biotechnology. Limnologica.

[66] Pikula K.S., Zakharenko A.M., Aruoja V., Golokhvast K.S., Tsatsakis A.M. (2019). Oxidative stress and its biomarkers in microalgal ecotoxicology. Curr. Opin. Toxicol..

[67] Balzano S., Villanueva L., de Bar M., Sahonero Canavesi D.X., Yildiz C., Engelmann J.C., Marechal E., Lupette J., Sinninghe Damstï J.S., Schouten S. (2019). Biosynthesis of long chain alkyl diols and long chain alkenols in *Nannochloropsis* spp. (Eustigmatophyceae). Pl. Cell Physiol..

[68] Sinetova M.A., Sidorov R.A., Medvedeva A.A., Starikov A.Y., Markelova A.G., Allakhverdiev S.I., Los D.A. (2021). Effect of salt stress on physiological parameters of microalgae *Vischeria punctata* strain IPPAS H-242, a superproducer of eicosapentaenoic acid. J. Biotechnol..

[69] Jónasdóttir S.H. (2019). Fatty acid profiles and production in marine phytoplankton. Mar. Drugs.

[70] Messner K.R., Imlay J.A. (2002). Mechanism of superoxide and hydrogen peroxide formation by fumarate reductase, succinate dehydrogenase, and aspartate oxidase. J. Biol. Chem..

[71] Quinlan C.L., Orr A.L., Perevoshchikova I.V., Treberg J.R., Ackrell B.A., Brand M.D. (2012). Mitochondrial complex II can generate reactive oxygen species at high rates in both the forward and reverse reactions. J. Biol. Chem..

[72] Quinlan C.L., Perevoschikova I.V., Goncalves R.L., Hey-Mogensen M., Brand M.D. (2013). The determination and analysis of site-specific rates of mitochondrial reactive oxygen species production. Meth. Enzymol..

[73] Brand M.D. (2016). Mitochondrial generation of superoxide and hydrogen peroxide as the source of mitochondrial redox signaling. Free. Radic. Biol. Med..

[74] Manhas N., Duong Q.V., Lee P., Richardson J.D., Robertson J.D., Moxley M.A., Bazil J.N. (2020). Computationally modeling mammalian succinate dehydrogenase kinetics identifies the origins and primary determinants of ROS production. J. Biol. Chem..

[75] Yakoviichuk O., Danchenko O., Kurtyak B., Nikolaeva Y., Fedorko A., Halko T. (2019). Ontogenetic features of redox reactions in the myocardium of Geese. Biologija.

[76] Rezayian M., Niknam V., Ebrahimzadeh H. (2019). Oxidative damage and antioxidative system in algae. Toxicol. Rep..

[77] Ma X., Deng D., Chen W. (2017). Inhibitors and activators of SOD, Gsh-Px, and CAT. Enzyme Inhibitors and Activators.

[78] Bhabak K.P., Mugesh G. (2010). Functional mimics of glutathione peroxidase: Bioinspired Synthetic Antioxidants. Acc. Chem. Res..

[79] Deng X.Y., Cheng J., Hu X.L., Gao K., Wang C.H. (2015). Physiological and biochemical responses of a marine diatom *Phaeodactylum tricornutum* exposed to 1-octyl-3-methylimidazolium bromide. Aquat. Biol..

[80] Wang T.Y., Libardo M., Angeles-Boza A.M., Pellois J.P. (2017). Membrane oxidation in cell delivery and cell killing applications. ACS Chem. Biol..

[81] Li X., Yang W.L., He H., Wu S., Zhou Q., Yang C., Zeng G., Luo L., Lou W. (2018). Responses of microalgae *Coelastrella* sp. to stress of cupric ions in treatment of anaerobically digested swine wastewater. Bioresour. Technol..

[82] Maltseva S.Y., Kulikovskiy M.S., Maltsev Y.I. (2022). Functional state of *Coelastrella multistriata* (Sphaeropleales, Chlorophyta) in an enrichment culture. Microbiology.

[83] Brigelius-Flohé R., Flohé L. (2020). Regulatory phenomena in the glutathione peroxidase superfamily. Antioxid. Redox Signal..

